# Platelet-derived concentrates influence human keratinocyte proliferation *in vitro* and induce wound healing in a prospective case series of chronic wounds of different entities *in vivo*

**DOI:** 10.1515/iss-2022-0011

**Published:** 2022-10-10

**Authors:** Anastasia Paulmann, Sarah Strauss, Anne Limbourg, Peter M. Vogt

**Affiliations:** Department of Nephrology and Hypertension, Medical School Hanover, Hanover, Germany; Department of Plastic, Aesthetic, Hand- and Reconstructive Surgery, Medical School Hanover, Hanover, Germany

**Keywords:** autologous platelet plasma, chronic wound, ease of surgical wound closure, laser Doppler imaging, PRP, wound healing, wound quality of life

## Abstract

**Objectives:**

Soft tissues defects can extend into the fat layer or even deeper and can cause significant clinical disadvantages like pain, infections, and loss of function. In particular, chronic wounds are difficult to treat, as split-thickness skin grafts (STSGs) have varying success rates. To improve wound healing in chronic wounds, the authors have studied the application of platelet-mediator concentrate (PMC) in a human keratinocyte culture model *in vitro* and of autologous platelet concentrates (PRP) in a combination with surgical procedures *in vivo* as second line therapy in patients with initially failed wound closure.

**Methods:**

For *in vitro* testing on keratinocytes, a PMC was processed with a commercially available bedside system (ATR^®^, Curasan, Germany). In a clinical, nonrandomized study, five in-house patients with chronic wounds were treated using a combination of surgical debridement and autologous PRP. Time of healing as determined by epithelization as well as laser Doppler imaging to visualize blood flow was analyzed. Additionally, changes in ease of surgical wound closure were determined. Finally, the quality of life of patients was assessed using a validated questionnaire (clinicaltrials.gov # NCT03667638).

**Results:**

*In vitro* testing shows a significant effect of PMC on keratinocyte proliferation in cell culture. Clinical studies showed that patients treated with PRP had initiation of wound closure, higher blood flow after PRP injection, and easier wound closure as well as improved quality of life.

**Conclusions:**

The injection of platelet concentrates to treat chronic wound defects presents a favorable addition to treatment where single surgical procedures have failed and may improve current therapy options.

## Introduction

Nonhealing wounds are still a huge clinical challenge. A chronic wound is one that has failed to progress through the phases of healing in an orderly and timely fashion and has shown no significant progress toward healing. Many factors can lead to such problematic wounds—for example, decreased blood circulation and bradytrophic wound margins. Currently, 1.5–2 million people in Europe are thought to suffer from chronic wound healing disorders. Chronic wounds have a significant impact on the patient’s quality of life, especially for the elderly [1]. Furthermore, chronic wounds represent a significant cost factor in the health-care system. This will continue to grow as a problem since the population of elderly suffering from predisposing diseases such as diabetes and obesity is increasing.

An acute wound usually undergoes three stages of healing: inflammation, proliferation, and remodeling [2]. In each of these stages, the cells comprising the wound secrete growth factors in different concentrations depending on the healing stage. The chronic wound on the other hand is arrested in the stage of inflammation [3]. Since the definition of when a wound becomes chronic varies between authors, a definition based on the appropriate timing of wound stage is more suitable. The authors’ hypothesis states that the induction of neovascularization, which plays a special role in the early stages of wound healing to provide the tissue with oxygen and nutrients, can take a chronic wound out of the arrested stage. Therefore, the interplay of epithelialization and angiogenesis has a major influence on the reversibility of a chronic wound.

Many experimental and clinical studies have shown a mostly beneficial effect from exogenous growth factors added to a standard therapy in acute treatment. Since wound healing is a complex interaction formed by a variety of growth factors, the effect of a single exogenous factor application has not been convincing in promoting the healing of impaired wounds [4].

Recently, the application of a growth factor cocktail derived from platelet concentrate was studied in acute wounds (such as burn wounds), and it shows promising results. To shed more light on the potential effects on chronic wounds of different entities, the authors examined the influence of platelet-enriched concentrates on keratinocytes in an *in vitro* assay and later in a pilot study of five chronic wounds of different entities. Chronic wounds were defined by a nonhealing wound state of over 21 days, refractory to surgical or conservative therapy, such as repeated surgical debridement or negative pressure wound therapy (NPWT). The aim of the study was to analyze the effects of platelet concentrates of chronic wounds, measured by healing rate as an expression of epithelization and the measurement of blood perfusion to analyze neovascularization.

Autologous platelet-rich concentrates can be roughly divided into two categories: platelet-rich plasma (PRP) and platelet-mediator concentrate (PMC). There are multiple methods to produce platelet concentrates [5]. Unfortunately, the lack of a standardized procedure to produce platelet concentrates remains a vexing disadvantage.

PMC is a mixture of already activated and lysed platelets; the growth factors are already in solution. The disadvantages include a shorter shelf life and efficacy and a low target concentration when compared to that of PRP at the desired site of action [6]. PRP, on the other hand, consists of intact platelets that have been concentrated fivefold on average [5]. In PRP, growth factors are released only upon tissue contact (e.g., with collagen fibers, as is typical in endothelial injuries) [7]. In addition, PRP is easier and less expensive to produce than PMC. With PMC, an additional activation step is needed to lyse the platelets—depending on the publication, the substances used differ, e.g., fibrin, calcium, or thrombin [8]. Platelet-enriched concentrates also have the advantage that the autologous manufacturing process significantly reduces the likelihood of transmission of transfusion diseases (such as hepatitis or HIV).

The PMC was used for *in vitro* testing. This consists of lysed platelets and their contents, i.e., it is a mixture of growth factors and platelet membranes. This was used because the cell culture lacks the target structures for physiological platelet activation. In the clinical part, on the other hand, intact platelets were considered more advantageous: during surgery, platelets can bind directly to the tissue and be activated there; therefore, PRP was used here. There are certified kits to produce PRP or validated procedures that enable sterile production during surgery.

Besides crude healing rates, the quality of life becomes increasingly important in wound therapy. It has been shown that chronic wounds lead to psychological distress even many years after the initial trauma [9]. Psychological distress can prolong the healing time [10]. In this study, the Wound – Quality of Life (W-QoL) questionnaire, developed in Hamburg, Germany [9], was used to evaluate the quality of life in patients before and after PRP injections. The W-QoL questionnaire is a tool for assessing wound-specific quality of life in patients with chronic wounds.

## Materials and methods

### *In vitro* testing

#### PMC preparation

The PMC was prepared by using the ATR Set^**®**^ (Curasan AG, Kleinostheim, Germany). This set uses a procedure combining sedimentation and filtration, instead of centrifugation. An optimal platelet and growth factor yield was demonstrated in the clinical studies [11]. Venous blood was taken from a healthy, 25-year-old female (who gave written informed consent prior to donating) using the included syringe. The PMC preparation was performed according to the instruction manual. To produce 2 mL of PMC, 8 mL of venous blood was needed. Altogether 2 ATR Sets^**®**^ were used for *in vitro* testing.

#### Keratinocyte culture and cell isolation

Primary keratinocytes were isolated at the Laboratory for Regeneration Biology of the Department of Plastic Surgery, Hanover, using the local protocol. Three patient samples were chosen: all female, aged 35–42 years, who underwent elective abdominoplasty. All patients provided their written consent according to the approval procedures of the ethic committee of the Hanover Medical School (#1055-2011).

Small pieces of tissue were prepared from full skin grafts under sterile conditions using scalpels and surgical tweezers on a clean room workbench and transferred to a 50 mL tube (TPP^®^, Trasadingen, Switzerland). The tube was incubated with a trypsin solution for about 10 min at 37 °C. It was then buffered using phosphate-buffered saline (PBS) w/o (without Ca^2+^/Mg^2+^). The cell mixture was centrifuged at 500 *g* for 5 min, the buffered solution was pipetted off, and the resulting pellet was resuspended with fresh medium. The isolated cells (10^6^ cells/mL, passage 0) were stored at −80 °C in 2 mL cryotubes for several weeks.

After thawing at room temperature, the cells were transferred to a 1.5 mL tube with PBS and then centrifuged at 220 *g* for 5 min at room temperature. The cells were seeded in three culture flasks (TPP^**®**^, Switzerland) per patient with 5 mL serum-free keratinocyte medium (PromoCell GmbH, Heidelberg, 0.06 mmol/L Ca^++^). The cells continued growing to confluence for a week with continuous manual medium changes every 3 days in a humidified atmosphere of 95% air and 5% CO_2_ in a tissue culture incubator at 37 °C.

#### Cell proliferation assay

The keratinocytes of each patient (10^4^ cells per well) were seeded onto 24-well plates (TPP^**®**^, Switzerland). The cells were cultured using a keratinocyte medium (PromoCell GmbH, Heidelberg, 500 µL per well) with two consecutively performed assays: 0% (control) and 1% (5 µL per well) PMC as well as another 0% (control) and 10% (50 µL per well) for 3 days. The purity of the keratinocyte cell type was confirmed using microscopy; if the total number of fibroblasts was higher than 10%, then the plate was disposed off. The PMC was added to each well every day during the time of measurement, whereas no medium exchange was performed. The times of measurement were 24, 48, and 72 h after seeding. The medium in the well was removed, and the cells were washed with PBS w/o. Subsequently, trypsin 0.05%/EDTA 0.02% (Cytogen^®^, Greven, Germany) was added to the cells and left on for about 2 min. The cell suspension was transferred to a centrifuge tube, diluted with PBS w/o, and centrifuged at 220 *g* for 5 min. The resulting pellet was resuspended in fresh medium, mixed with a pipette, and diluted with trypan blue solution to a final concentration of 0.5%. Living cells (seen as light reflecting) were counted manually using a Fuchs-Rosenthal counting chamber under a light microscope (Olympus).

#### Cell migration assay/scratch assay

For cell migration assays, the keratinocytes (1.5 × 10^5^ cells per dish) were seeded onto 22 cm^2^ dishes (TPP^**®**^, Switzerland) with 15 mL of keratinocyte medium per dish. After the cells became confluent, a scratch using the tip of a micropipette was set—the scratch was approximately 400–450 µm wide. After washing, a new medium was added including PMC—1% PMC dishes were added with 15 µL of PMC, whereas 10% dishes had 150 µL of PMC. On each dish, three areas were marked to determine the width of the scratch. Combined migration and proliferation of cells was observed using an inverted microscope (Zeiss Axiovert) 0, 12, 24, 36, and 48 h after scratching.

#### Statistical analysis

Averages of three patients with each three replicates (n=9) are presented as mean ± 1 standard deviation. A statistical significance between PMC and control values was determined using a one-way ANOVA test (*α*=0.05) in Microsoft Excel^®^.

### *In vivo* testing

All patients provided their written consent according to the approval procedures of the ethic committee of the Hannover Medical School (# 3152-2016). The study was registered on clinicaltrials.gov (# NCT03667638). The Agency for Healthcare Research and Quality finds all case series to have a Level of Evidence 5, whereas in the definition of the Centre for Evidence-Based Medicine, all case studies are Level 4.

#### Patients’ including and excluding criteria

**Figure 1:**
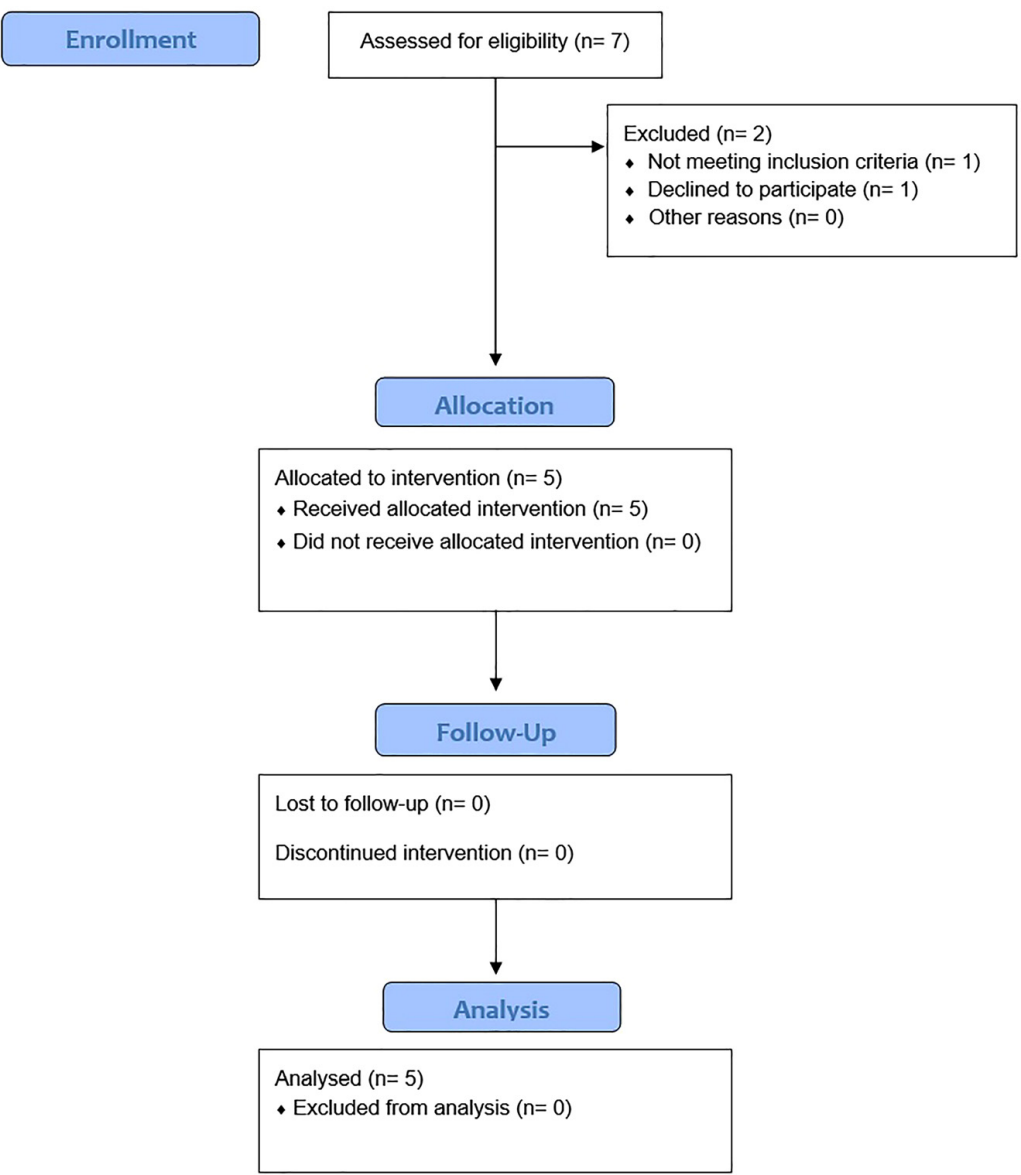
Consolidated standards of reporting trials (CONSORT) flow chart for the intervention with PRP.

A prospective case series with five chronic wounds was performed. A chronic wound was defined by a nonhealing wound state of over 21 days, refractory to surgical or conservative therapy. The chronic wounds analyzed included exposed tendons, ligaments, or bone (see Table 1). Participants (mean ± SD aged 65.4 ± 14.3 years) were selected as in-house patients in the Department of Plastic-, Aesthetic-, Hand and Reconstruction Surgery of the Medical School, Hannover, during the period from November 2016 to November 2017. All patients who volunteered were informed about indication, possible side effects, and study design and had signed a written informed consent. Exclusion criteria included sepsis criteria, continuous smoking, pregnancy or breastfeeding women, and participants of other studies (Figure 1).

**Table 1:** Overview of patients treated with PRP.

Patient number	Gender and age, years	Wound description	Previous procedures and timely course (see Figure 6)	Comorbidities	Days in hospital
°1	Male, 68	Large wound with undermined wound margins after gluteal abscess	100 days prior to PRP:	Hypertension, chronic renal insufficiency, and diabetes	63 days
			abscess resection		
			13× debridement		
			no NPWT		
°2	Male, 71	Diabetic foot ulcer, state after toe amputation	22 days prior to PRP:	Hypertension, COPD, and diabetes	55 days
			Amputation		
			5× debridement		
			NPWT		
°3	Male, 79	Diabetic foot ulcer, state after fore foot amputation	47 days prior to PRP:	Heart disease, hypertension, chronic renal insufficiency, and diabetes	53 days
			Amputation		
			7× debridement		
			NPWT		
°4	Male, 44	Duodenal fistula after severe burn	140 days prior to PRP:	Burn wound area approx. 40%, fatty liver disease, and hepatitis C	287 days
			27× debridement		
			NPWT		
°5	Male, 75	Venous foot ulcer	23 days prior to PRP:	Polycythemia vera, hypertension, heart disease, and hyperlipidemia	38 days
			Amputation		
			5× debridement (only 2× in our clinic)		

All subjects suffered from long-standing wounds and were submitted to surgical wound closure. Since previous surgery had failed, the use of PRP in conjunction with surgical wound closure was chosen. NPWT, negative pressure wound therapy.

#### PRP preparation

The PRP was used for the *in vivo* testing. For the first patient, a commercially available kit (Yes PRP^**®**^, RegenAging, Austria) was used to prepare the autologous PRP. Later, a manual preparation method was employed; hence, patients 2–5 received manually prepared PRP. Manual PRP preparation followed a two-step process. A small volume of blood (20 mL) was collected from each patient during surgical wound debridement through a venous catheter. In the first step, whole blood was centrifuged for 10 min at 2400 rpm (950 *g*) to separate the red blood cell portion; in the second centrifugation step for 15 min at 3600 rpm (2,200 *g*), the PRP was separated from the platelet-poor plasma [12] at room temperature. This amounted to approximately 10 mL of PRP. The PRP was then transferred to a sterile syringe and given to the operating room nurse until injection.

#### Wound preparation and split-thickness skin graft harvesting

**Figure 2:**
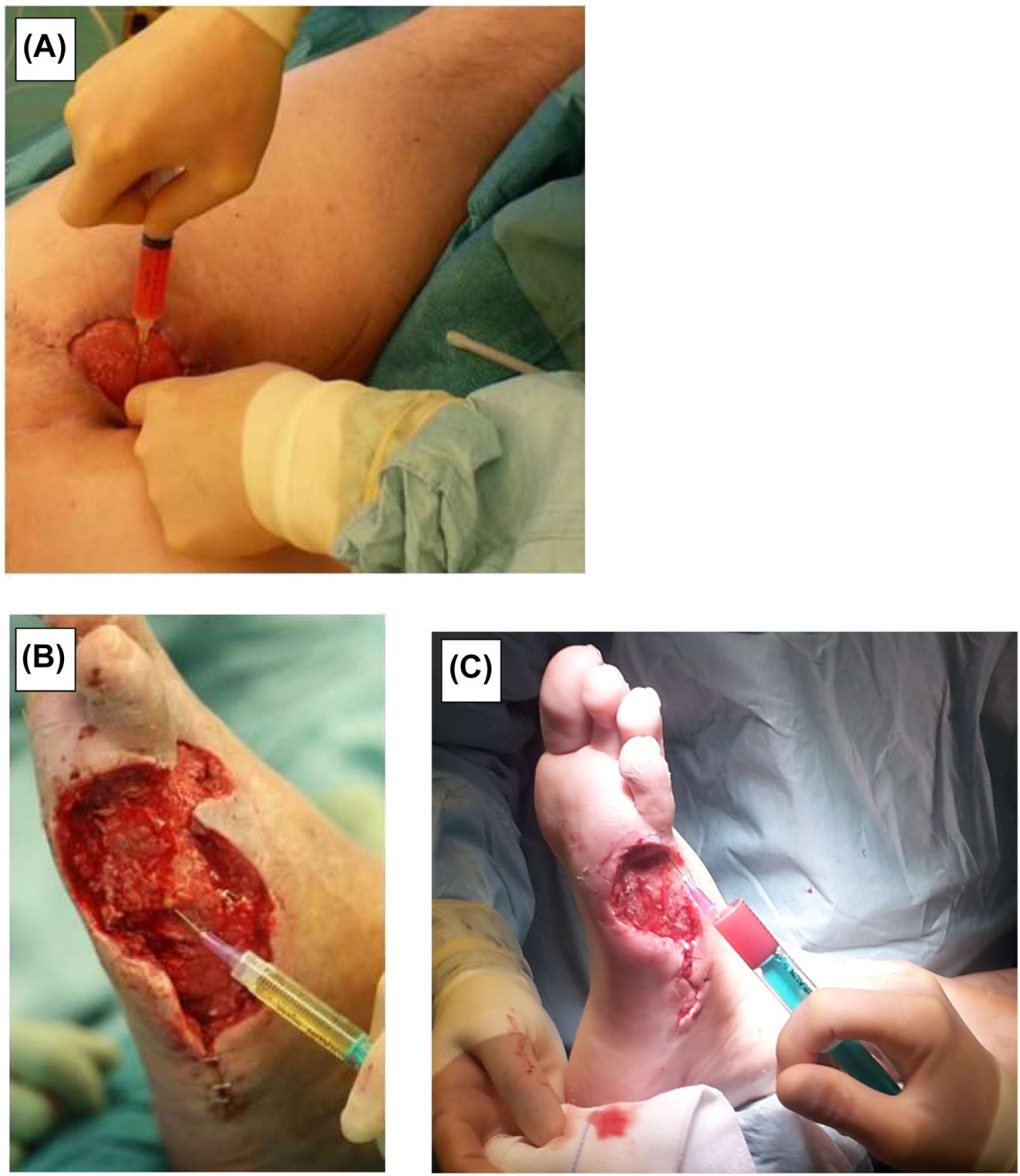
Examples of PRP injection in patient °1 (A) as well as patient °2 (B + C). Injections into wound base at day 0 (B) and wound margin (C) at day 14. All wounds had PRP injection into wound margins and base every time.

Each patients’ wound was treated initially by mechanical surgical debridement and extensive cleansing using 0.9% saline to remove all necrotic or potentially infectious tissue. The wounds were injected with the prepared PRP (10 mL) using a sterile syringe with a 22-gauge needle into the wound margins as well as wound base to cover as large an area as possible. The wound was then covered with foam dressing, which stayed in place for 3 days to ensure manipulation free healing. The PRP application was performed on days 0, 7, and 14. If coverage of the wound was possible (when granulation tissue was present), split skin grafts were harvested from the anterior thigh using a Padgett dermatome, approximately 0.2 mm in width, and secured in place using staples. Examples of injection are shown in Figure 2.

#### Follow-up care

##### Primary outcome measure

**Figure 3:**
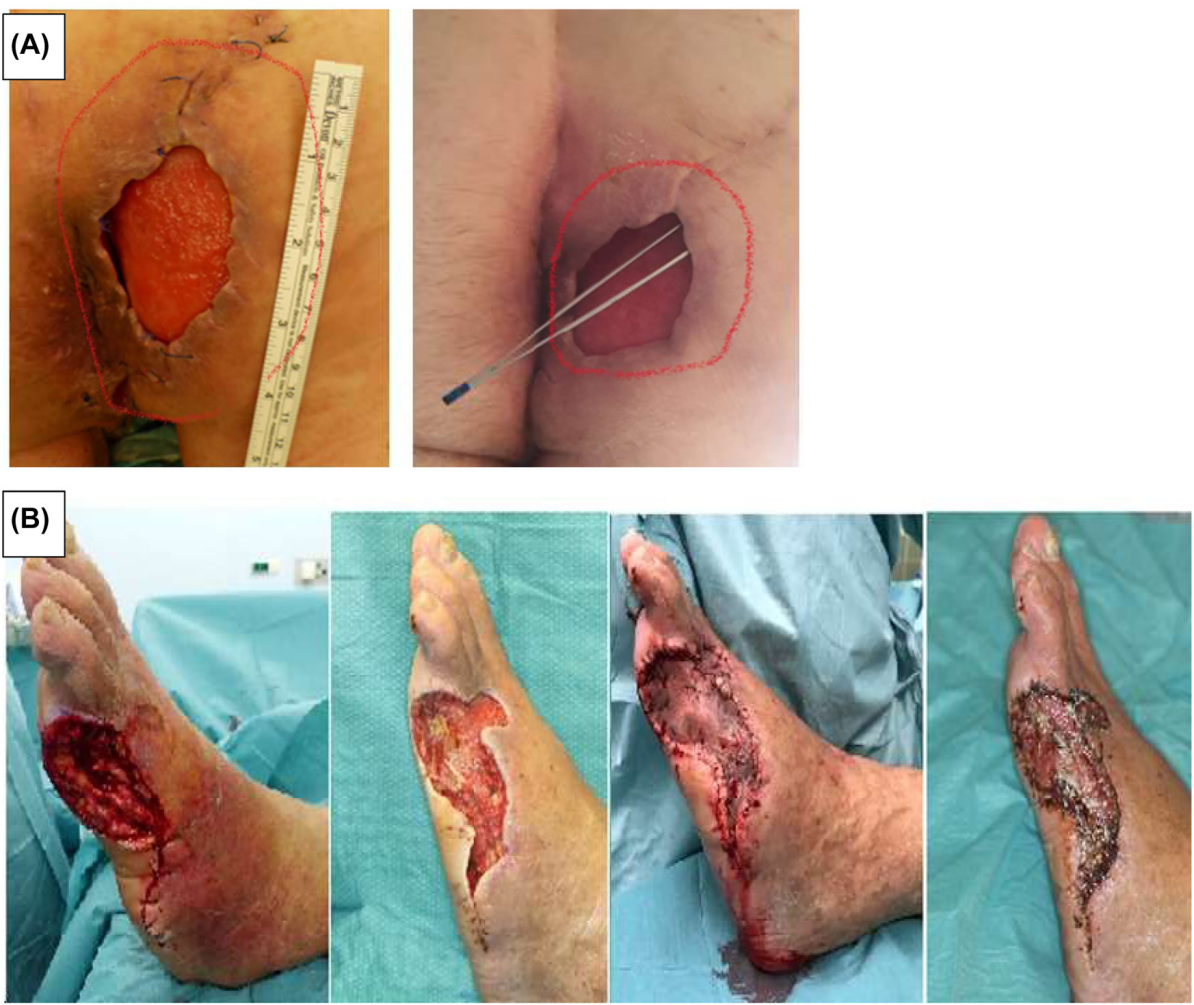
Examples of wound healing in patient °1 (A) and patient °2 (B). The first row shows patient °1 had large undermined wound edges (first picture on day 0), which greatly decreased after PRP injection (second picture on day 21). The second row (B) shows patient °2 on days 0, 7, 14, and 21. The wound area reduced after STSG due to contraction.

Wound healing was documented using digital photography on days 7, 14, and 21 post-surgery during dressing changes. Hospital stays varied between 53 and 287 days. It should be noted that patient °4 had significantly longer hospitalization than the other patients. Wound area was calculated using Adobe Photoshop^®^. We used retrospective data from surgery protocols and previous photo documentation to reconstruct the wound area development over time. Examples of healing are shown in Figure 3.

##### Secondary outcome measures

**Figure 4:**
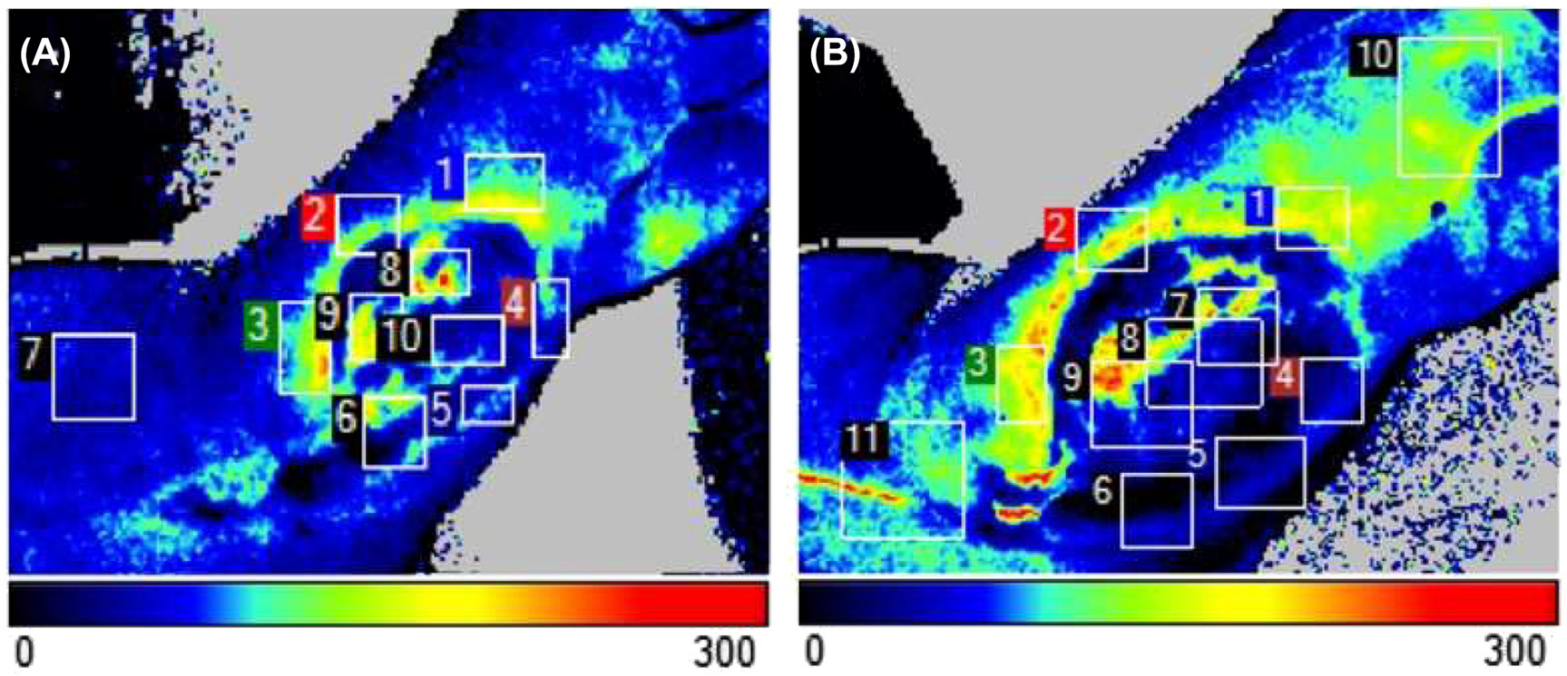
LDI of patient 5 on day 0 (A) and day 7 (B). Shown are measurements of wound margin, wound base, and regular skin. Perfusion is shown as scale in Perfusion Units (0–300 PU): light gray/white equals low blood flow, black/dark equals high blood flow. Picture B has higher perfusion in wound ground and margin, indicating an angiogenetic effect after the injection of PRP.

Laser Doppler imaging (LDI) was performed before surgery and on days 7, 14, and 21 post-surgery to analyze the vascular state of the wound. The device used was a PeriCam PSI System (PeriMed, Sweden) with associated software. The device had a mobile scan head, which allowed appropriate positioning relative to the patient. The distance between the scan head and the wound was approximately 20 cm. The scan was performed at room temperature in a darkened room during the routine dressing change; the adjacent skin was covered by sterile dark green cloth to minimize light reflection. The patient was asked not to move or talk during scans and was not talked to in order to minimize artificial blood flow. We performed one measurement on regular skin, three measurements on the wound base, and six measurements on the wound margin in a circular fashion; the initial measurements were then averaged (see Figure 4).

Before surgery as well as on day 21 post-surgery, the quality of life was assessed using a standardized and validated questionnaire, the W-QoL questionnaire (University Medical Center Hamburg-Eppendorf, Germany), following the instructor’s manual. The W-QoL questionnaire is a tool for assessing wound-specific quality of life in patients with chronic wounds. It consists of 17 items divided into subscales (“body”, “psyche”, and “everyday life”) on impairments which are always assessed in retrospect to the preceding 7 days [9].

The ease of closure score (see Table 2) was evaluated on day 0 as well as on day 21 (beginning and end of study period) from photographs.

**Table 2:** Ease of closure score, modified from Robson et al. [13].

Score	Treatment	Costs (in $)
0	No need to close, healed	0
1	Easy wound approximation	100–400
2	Somewhat easy wound approximation	400–600
3	Somewhat difficult wound approximation	600–800
4	Difficult wound approximation	800–1,000
5	Easy skin graft	1,200
6	Somewhat easy skin graft	1,700
7	Somewhat difficult skin graft	2,200
8	Difficult skin graft	3,000
9	Easy flap	5,000
10	Somewhat easy flap	8,000
11	Somewhat difficult flap	10,000
12	Difficult flap	12,000
13	Not possible to close	Not determinable

## Results

### *In vitro* testing

#### Cell proliferation assay (n=3)

**Figure 5:**
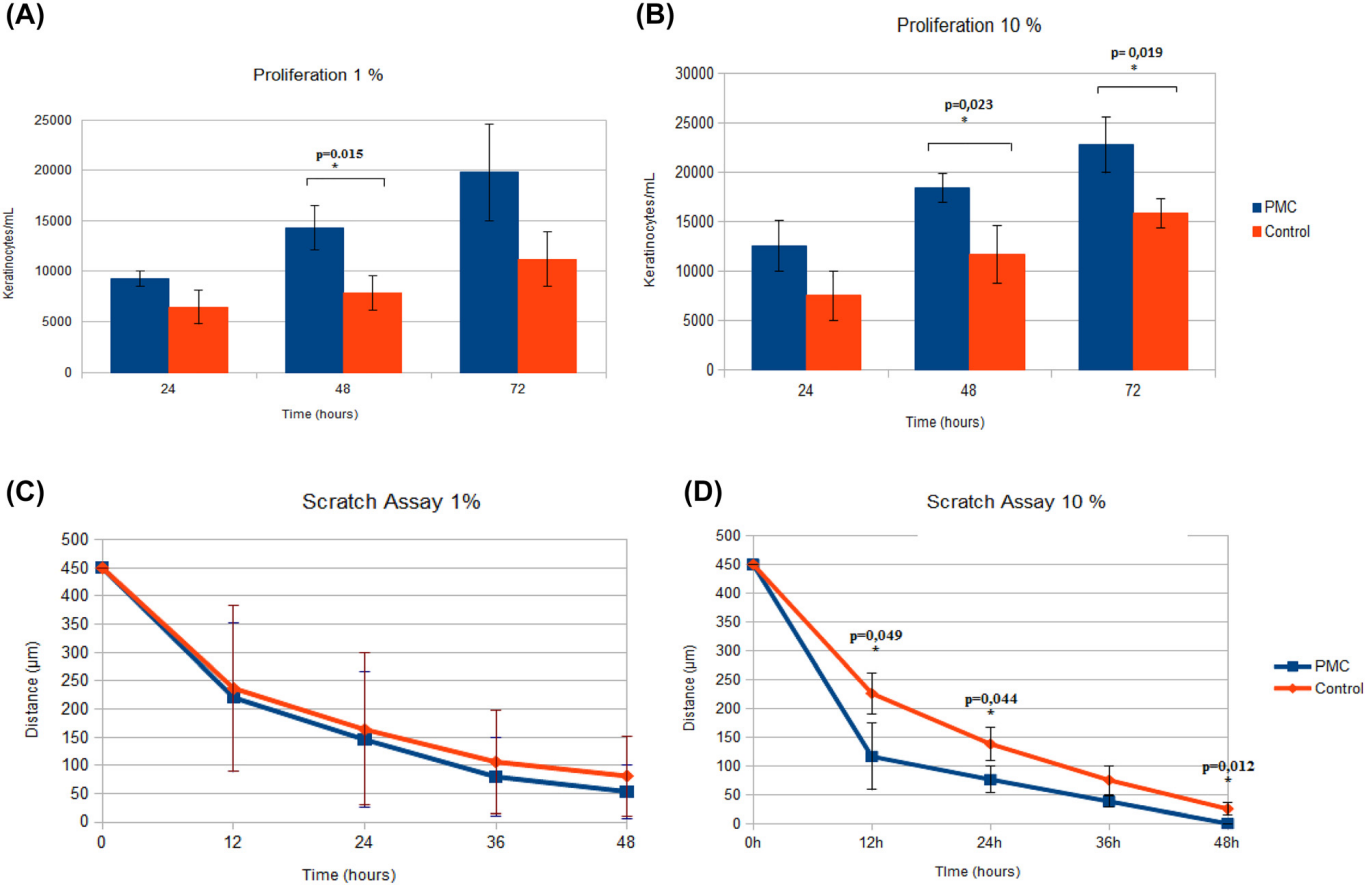
Keratinocyte culture assays using PMC. (A and B) Proliferation assays using 1 and 10% PMC. Higher concentration of PMC shows higher proliferation rates with significant differences after 48 and 72 h. (C and D) Scratch/migration assays using 1 and 10% PMC. PMC effect on proliferation and migration is also concentration dependent.

Cell proliferation was enhanced as early as 24 h after incubation when compared to controls (Figure 5A and B). This positive effect increased significantly after 48 h and persisted up to 72 h. Higher concentrations of PMC seem to cause a stronger proliferation than 1% PMC, as results after 48 and 72 h show a significant difference compared to the control. In contrast to the low concentration, the difference in 10% PMC was statistically significant after 72 h also. The absolute cell numbers are also higher than that in lower concentrations. After the first seeding and PMC administration, the number of cells seems to be reduced. Only after 48 h, the cells show measurable differences in proliferation rates.

#### Cell migration assay/scratch assay (n=9)

Our scratch assays show a combination of migration and proliferation of keratinocytes in cell culture after the addition of PMC (Figure 5C and D). Using 1% PMC in the scratch assay does not show any significant difference to the control group. On the other hand, using 10% PMC shows a significant difference from the beginning. This correlates with the results above.

### *In vivo* testing

An overview of patients included in this study is shown in Table 1. The results are shown for each patient as well as an average (mean ± SD) for primary end point, the wound area (in mm^2^), secondary end points, blood flow measured on wound base, wound margin, intact skin (control), and finally quality of life. Due to the significant *in vitro* effects of 10% PMC, the same concentration was also used in PRP for the patient study.

#### Wound area and ease of closure

##### Patient overview and history

**Figure 6:**
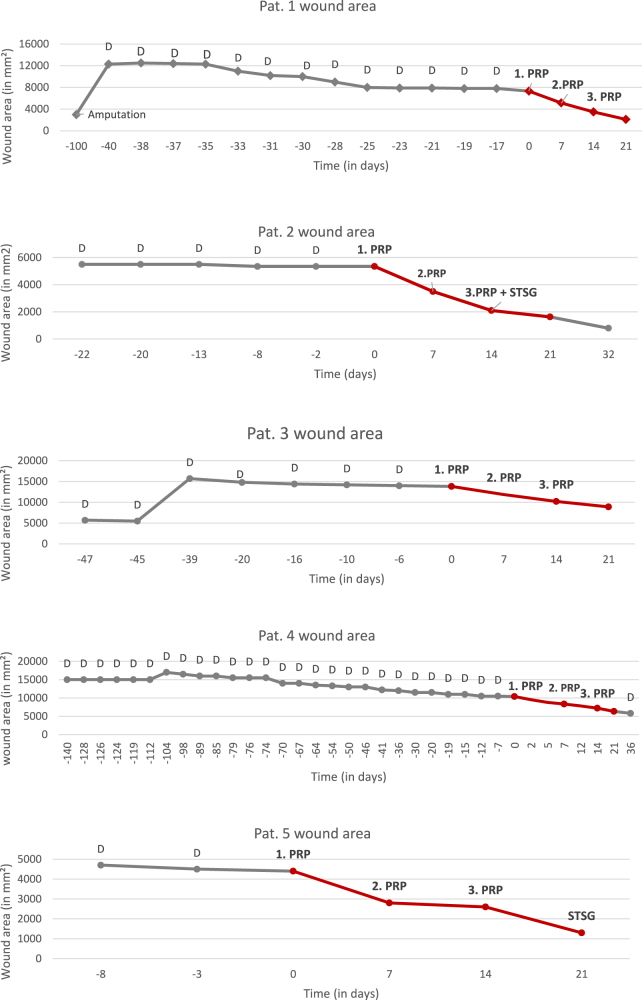
Wound areas of all patients in mm^2^ over time. All patients had arrested wound healing before. After injection with PRP, a significant reduction in wound area was observed. D, debridement; PRP, platelet-rich plasma; STSG, split-thickness skin graft.

Each patient’s data are documented as wound area in mm^2^ over a time period in days (Figure 6). We began counting the days from the time of study enrollment (days 0–21, black). Retrospective data were used to show the wound area development over time (gray). The PRP injections during surgery are shown as “PRP” above time points. Surgical debridement is abbreviated with “D”. The ease of closure score was determined on days 0 and 21.

##### Data summary

Our data show that 3 weeks after the first PRP injection, the wound area was reduced by 42 ± 19%. By comparing each time point to the previous one, we were able to show a significant reduction within the first weeks after the first PRP injection in wounds that did not show proper healing rate previously. Although PRP was applied at day 7 and 14 as well, there is no statistically significant reduction in wound area. Patients 1, 2, and 5 all had smaller wound areas (<10,000 mm^2^), and their wounds show an even higher healing rate of 27.9 ± 2.5% (p-value <0.0001) after 21 days when compared to the overall analysis.

**Figure 7:**
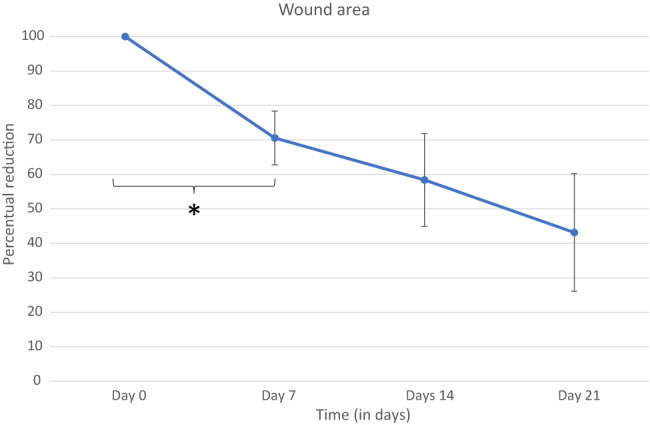
Average wound area in percentage (%). After PRP injection, there is a reduction in wound area. This effect is statistically most significant after the first injection. Data are compiled from 5 patients. *: p-value <0.05.

**Figure 8:**
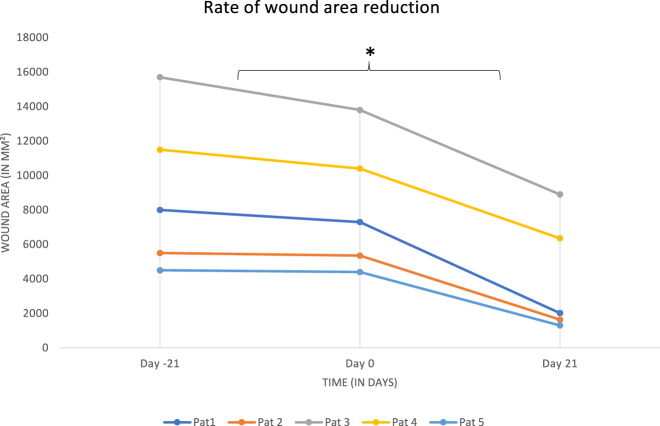
Rate of wound area reduction. After PRP injection, there is a significant change in the rate of healing, *: p-value <0.05.

All measurements of wound areas and an average are shown in Figure 7. Data are shown in percentage starting from the initial wound area for better comparison. Figure 8 shows the change in healing rate after PRP injection for each patient over time.

The ease of wound closure measurement is shown for each patient as well as an average (Table 3). Our data show an average ease of closure score of “10” on day 0. After 3 PRP injections, this score was significantly reduced to “3” on day 21 (p-value=0.00134).

**Table 3:** Ease of wound closure scale on day 0 and day 21.

Patient	Day 0	Day 21	Improvement on scale
Pat. #1	12	3	9
Pat. #2	8	0	8
Pat. #3	11	3	8
Pat. #4	13	6	7
Pat. #5	8	5	3
**Average (Mean ± SD)**	**10 ± 2**	**3 ± 2**	**p-value=0.00134**

After PRP treatment, a significant reduction in the ease of wound closure is achieved.

#### Perfusion

**Figure 9:**
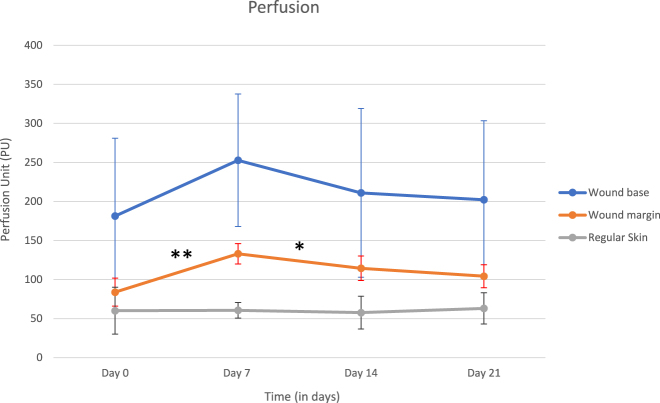
After PRP injection, there is a difference in perfusion in the wound base and especially in the wound margin. This change in perfusion is most visible after the first PRP-injection. Data are compiled from 5 patients. **: p-value <0.01. *: p-value <0.05.

Our data show that before the first PRP injection, a chronic wound can be divided into two major areas concerning perfusion: wound base and wound margin (Figure 9). Compared to healthy skin (no visible damage), the perfusion of the wound base is higher from the start. On the other hand, wound margin had less perfusion when compared to the wound base.

When compared to the first time point (day 0), we were able to show a significant improvement in wound margin perfusion on day 7 and day 14 (day 7, p-value=0.004715 and day 14, p-value=0.033189). After 21 days, there is no significant difference in perfusion when compared to day 0. There is an impact on the perfusion of wound base after using PRP, though it is not statistically significant.

#### Wound-Quality of Life

All patients were interviewed on day 0 and day 21 using the W-QoL questionnaire (Table 4).

**Table 4:** Quality of life as measured with W-QoL questionnaire.

Patient number	Day 0	Day 21	Day 21 interview in …
Patient °1	1.8	1.4	Outpatient clinic
Patient °2	2.7	1	Outpatient clinic
Patient °3	2.9	1.9	Hospital
Patient °4	3.4	3.2	Hospital
Patient °5	2.5	1.3	Hospital
**Average**	**2.6**	**1.7**	

An improvement in the quality of life was shown after 21 days.

The results can be interpreted as follows: a value of “0” would mean no effect from a wound on daily life, mental state, or body integrity. A value of “4” would mean a severe effect on these indicators. A value of “2” would correspond to moderate effect. In summary, the higher the value, the worse the wound-related quality of life would be.

All patients started with a relatively high value, meaning a low quality of life and a high subjective complaint. After 21 days of treatment, some patients showed promising healing rate and were discharged from hospital care, whereas others still had to stay. The result after 21 days depended on whether the patient was discharged from the hospital or was still under treatment. Although aspects such as pain, odor from the wound, or wound secretion became less frequent, the psychological impact of the long hospital stay began to show (higher rating in “feeling depressed”, “frustration”, and “worry”). Once again, a subanalysis was made for the “fitter” patients 1, 2, and 5, who had an average quality of life of 1.23 after 21 days (p-value=0.0210), whereas the “sicker” patients 3 and 4 remained hospitalized even after the study was completed.

## Discussion

The use of platelet concentrates has grown in various other fields of medicine, such as ophthalmology, periodontology, and plastic surgery. It is used for wound treatment of soft tissue defects (e.g., ulcers) or bone regeneration [3]. The idea to use platelet concentrates to improve wound healing has been around since the 1980s. First successful trials using platelet-derived growth factors (PDGFs) were performed in 1996.

Platelets contain a massive amount of growth factors in their alpha granules, for example, PDGF and transforming growth factor beta, as well as anti-inflammatory and proinflammatory cytokines, tumor necrosis factor-α, and interferon-α [7]. Others are angiogenesis promoting growth factors, such as endothelial growth factor (EGF) and vascular endothelial growth factor (VEGF) [14]. For example, PDGF alone was shown to stimulate wound healing in randomized clinical trials. These stimulating growth factors have reduced endogenous levels in chronic wounds [6].

It can be concluded that chronic wounds lack the factors to proceed from the inflammatory to the proliferation stage, and that by adding growth factors externally, this step can be induced [15]. However, the healing process operates through a variety of factors and their cooperation involving more than one pathway [16]. Thus, reducing the stimulation potency of a mixture of growth factors to a single pathway would not reflect its true function [17]. Stable proliferation and angiogenesis are rather achieved by a physiological ratio in different growth factors, as it can be found in platelet concentrates.

There is no uniform definition on the time period of when a wound is considered chronic. An appropriate guidance can be given by the stages of healing: inflammation, proliferation, and remodeling [2]. In the case of chronic wounds, the periods of a phase can vary greatly and vary depending on the patient, wound cause, and wound condition. As stated above, we used a definition of >3 weeks of nonhealing state and being refractory to treatment. Chronic wounds can occur from various entities such as venous leg ulcers, diabetic foot ulcers, or arterial insufficiency and the combination of various factors. The treatment of chronic wound crosses many disciplines—this may be another reason why there is no common definition [1].

There are two main types of platelet concentrates: PRP and PMC. This differentiation is not widely employed. In some cases, investigators refer to PRP, although PMC was used. PMC is a solution of activated and lysed platelets, which already released their growth factors. Therefore, the disadvantages include its short life span and inefficient local delivery to target cells [6]. On the other hand, PRP is a solution of whole platelets in concentrations fivefold above the baseline [5], which releases its growth factors upon contact with human tissue [14], respectively, collagen after endothelial injury [7]. This is a more physiological pathway. Furthermore, it was shown that the preparation of PRP is easy and cost-effective when compared to the preparation of PMC, which is more time-consuming since it requires an additional step of activation. In addition, the substances used for activation differ between authors, for example, fibrin, calcium, or bovine thrombin [8]. Since platelet concentrates are made using autologous blood, the risk of disease transmission of HIV or hepatitis as it could occur in other blood products, is significantly reduced.

The complex wound repair process can be susceptible to a variety of pathologies in numerous healing phases. For its understanding, it is essential to compare the healing process of an acute wound to a chronic wound. The repair process in acute wounds consist of overlapping steps: inflammation, proliferation, migration, and contraction as well as angiogenesis [1]. Each phase requires different cell types, for example, platelets, fibroblasts, or monocytes. Additionally, a vast diversity of growth factors plays a role in wound healing. These growth factors can be concentrated twofold to threefold in platelet concentrates [18].

Impaired wounds have been shown to have less fibroblast and keratinocyte proliferation due to poorer responses to regular levels of growth factors, for example, PDGF. Chronic wounds are, therefore, arrested in the inflammation phase of wound healing. Surgical debridements are required to induce the wound from its quiescence state into a state of active proliferation. Previous studies have shown that different types of growth factors are present in varying concentrations in phases of wound healing [19]. For example, in the early healing stages, growth factor and cytokines released by platelets regulate proliferation and migration of keratinocytes, and therefore play a major role in shifting from inflammation to the phase of proliferation [20]. In later stages of wound healing, factors derived from monocytes and fibroblasts are necessary for proper wound development. Cho et al. showed that platelet concentrates may influence senescent fibroblast cells through enhanced expression of cyclin A, CKD4, and cyclin E to accelerate the healing process [21].

Neovascularization is important in the early stages of wound healing because early growth factors from platelets diffuse through the edges of blood vessels into the wound bed and induce proliferation. In particular, elderly skin was shown to have a greater proportion of tissue hypoperfusion, which leads to slower healing rates. “Sprouting” of new blood vessels into the new wound margins is also necessary for nutritive and oxygen supply [22]. Most prominent effects are caused by VGEF. The VGEF not only promotes angiogenesis, but also controls the vascular permeability for other cell types [23]. In summary, the interaction of epithelialization and angiogenesis plays an important role for the reversal of a chronic wound state.

To improve wound healing in such chronic wounds, we analyzed the additional application of autologous platelet concentrates in a keratinocyte culture model *in vitro* and in chronic wounds during surgical procedures *in vivo*. It served as an exogenous “booster” for endogenous healing signaling. Our primary goal was wound closure. Since wound repair in chronic large and complex wounds is a time-consuming process, 100% wound closure is not always an achievable clinical outcome. Determining the effect of a growth factor added to a surgical procedure can be a problem. Although for small wounds, 100% wound closure is a standard outcome, larger and more complex wounds can have another acceptable outcome to determine its clinical significance. For this purpose, Robson et al. proposed the use of an ease of closure scale [13]. The intention of this score is to evaluate the efficacy of a new method by reducing the complexity of necessary surgery. The reduction in ease of closure score correlates with decreased treatment costs.

Furthermore, we used LDI to quantify the blood flow inside the wound and its margin. LDI is a noninvasive method which uses the Doppler principle to measure and visualize the perfusion of superficial cutaneous tissue [24]. A laser light is directed at moving red blood cells; the reflected light is then used to calculate wavelength change which is proportional to the perfusion in the tissue. The unit of measurement is Perfusion-Unit (PU). The LDI displays a false-color image in which the high blood flow areas are colored red, whereas the low blood flow areas are blue, independent from the blood flow direction. Blood flow measured using LDI was shown to correlate with wound healing time and is especially useful in combination with digital photography to analyze microvascular blood flow in burns, scars, and ulcers [25].

Finally, we evaluated the quality of life of patients in terms of wound impact.

In summary, the results indicate that platelet concentrates can lead to an initially faster proliferation and migration of keratinocytes *in vitro* depending on the applied concentration of the platelet concentrate. The platelet concentrate used for *in vitro* testing was \platelet-mediator concentrate (PMC), which is a mixture of lysed platelets that already released their growth factors. Keratinocyte growth is especially accelerated by EGF-family growth factors and PDGF. Higher concentrations of platelet concentrate on keratinocytes have been shown to enhance inflammation instead [26]. This effect is important in acute wounds: after release of growth factors by platelets, the keratinocytes migrate into the wound from the wound margins, thus primary closure is achieved. Chronic wounds heal mostly through secondary closure. Another difference is that in acute wounds, the growth factors diffuse into the wound margins through capillaries, which are not present in sufficient amount in chronic wounds.

The results of *in vivo* testing show a reduction of wound area and an increase of blood flow into areas injected with PRP. The study is limited to a small number of patients (n=5) with heterogenous wound entities, which was ultimately due to the recruitment directly from the daily clinical routine. The original causes of the wounds (such as venous congestion or inadequate diabetes treatment) have been treated prior to study recruitment; however, the wounds remained even after resolving the triggering causes. All patient recruited for the *in vivo* study were males, whereas *in vitro* experiments were performed on female cells. Older men were shown to have significantly slower healing rates than women of the same age, due to positive regulatory effects of estrogen [27]. There was no comparison group due to ethical reasons, as this pilot study was designed as individual healing attempt for each patient.

To transfer the most efficient *in vitro* protocol into the clinical situation, patients with long-standing and heterogeneous wounds were chosen. Since they all showed arrested wound healing, as documented by multiple failed surgical debridement and inadequate wound healing after negative pressure therapy, they were ideal candidates to study the change in ease of surgical closure. The most striking effect was that unlike *in vitro* testing—where growth of keratinocyte was improved by higher concentrations of platelet concentrate in the medium—the *in vivo* testing shows the most significant difference only after the first platelet concentrate injection (between days 0 and 7). This can be explained by reviewing the physiological healing process in acute wounds: PDGFs play a major role only in the initiation of wound closure. After activation, monocytes and fibroblasts start releasing their growth factors, which then lead to matrix differentiation and keratinocyte proliferation/migration. With PRP, we were able to take the wound out of the arrested, inflammatory state and induce a more physiological healing process. After this initial induction, no further PRP was necessary, since then, other nonplatelet-related growth factors are the key players. The best effect of the platelet concentrate was detected on adherent wound corners, such as undermined wound margins. Most of the wound area reduction effect was derived from wound corners merging or a strong reduction in wound area by contraction of the wound margins. This is in line with our findings that the wound margins benefit the most from platelet injection by sprouting of small new vessels as in primary healing. On the other hand, the PRP addition to chronic wounds cannot be compared to that of acute wounds. One hypothesis was that the concentration level of growth factors at different time points are relevant for proper signaling, thus levels too high and too low (as in chronic wounds) impair wound healing [28]. Taken together, successful wound closure depends on the optimal balance of growth factor levels. Another limitation to this pilot study was that due to the limited cases, a general conclusion about the PRP effects on specific wound etiologies cannot be made. Certainly, further studies, especially for specific entities, would be interesting and would further specify the clinical use of PRP.

Blood flow measured by LDI was accelerated after the first and second PRP injections. This effect is mostly due to neovascularization in the wound margins. Since platelet concentrates contain angiogenesis promoting factors, such as VGEF and angiopoietins, Ang1 and Ang2, this was an expected effect. Results suggest that platelet concentrates contain a physiological ratio of growth factors that mimic the initial wound closure signaling, transforming the inflammation state of chronic nonhealing wounds into the first proliferation state. The mechanism by which platelet concentrates contribute to angiogenesis is still unknown. Mammoto et al. suggested that the platelet concentrate rich in Ang1 promotes angiogenesis by the Tie2-pathway in mice [17]. Another possible pathway is by VGEF signaling, which was shown to have a predominant role in angiogenesis. Improved angiogenesis results in better wound healing independent of the effect on keratinocyte proliferation/migration, since better vascularization leads to higher levels of endogenous growth factors in the wound. Our results suggest a better angioproliferative effect in smaller wounds. This is most likely due to a concentration difference. All patients received 10 mL of 10% concentrated PRP by injection, independent of the wound area. This leads to a dilution of growth factors in larger wounds, which then prevents a proper angioproliferative signaling in all the wound corners. The method of using laser Doppler to quantify blood flow requires experienced personnel and cannot be performed without previous training or supervision. To minimize operating bias such as patient blood pressure, positioning, and body or extremity temperature, we averaged most of our measurements into one representative measurement.

No studies regarding the effect of platelet concentrates on the quality of life were made so far. Our finding that the quality of life is associated with the extent of the wound is in line with previous studies. Chronic wounds severely lower the quality of life. On the other hand, psychological distress, such as depression, anxiety, and hostility, have a clinically relevant impact on wound healing [10]. In our study period of 3 weeks, we found an improvement in the quality of life over the course of treatment, but the quality of life was not completely restored. Additionally, a psychological effect of PRP may be due to its autologous properties with almost no side effects as a “drug”, and therefore may be better accepted by the patient.

## Conclusions

In summary, this work shows how platelet-rich concentrates have a positive effect on the healing of chronic wounds. This effect is mediated by an increase in the proliferation of keratinocytes, as could be shown in the *in vitro* tests, and the improved blood flow to the edges of the wound. This leads to a simplification of the surgical wound closure and consequently to a better quality of life for the patients.

## Supplementary Material

Supplementary Material
