# Supplementary material for: Platelet-derived concentrates influence human keratinocyte proliferation in vitro and induce wound healing in a prospective case series of chronic wounds of different entities in vivo
Source: Innov Surg Sci. 2022 Oct 10;7(2):45–58. doi: 10.1515/iss-2022-0011 (PMC9574654; doi:10.1515/iss-2022-0011)
Supplement: Supplementary file 1 — Supplementary Material [file j_iss-2022-0011_suppl.pdf]

Reviewer Assessment

A. Paulmann et al.: Platelet-derived concentrates influence human keratinocyte proliferation in vitro and induce wound healing in a prospective case series of chronic wounds of different entities in vivo

Reviewers' Comments to Original Submission

Reviewer 1: Prantl, Lukas

Date received: 17-May-2022
Reviewer recommendation: **Return to author for minor modifications**
Reviewer overall scoring: **High**

Assessment Form scores: 5 = High/Yes; 3 = Medium/Adequate; 1 = Low

Is the subject area appropriate for the journal		4			
Does the title clearly reflect the paper's content?			3		
Does the abstract clearly reflect the paper's content		4			
Do the keywords clearly reflect the paper's content?	5				
Does the introduction present the problem clearly?		4			
Are the results/ conclusions justified?	5				
How comprehensive and up-to-date is the subject matter presented?		4			
How adequate is the data presentation?		4			
Are units and terminology used correctly?		4			
Is the number of cases adequate?			3		
Are the experimental methods/ clinical studies adequate?		4			
Is the length appropriate in relation to the content?	5				
Does the reader get new insights from the article?		4			
Please rate the practical significance.		4			
Please rate the accuracy of methods.		4			
Please rate the statistical evaluation and quality control.	5				
Please rate the appropriateness of the figures and tables.	5				
Please rate the appropriateness of the references.		4			
Please evaluate the writing style and use of language.		4			
Please judge the overall scientific quality of the manuscript.		4			

Are the methods used worthy of reproduction in greater deal?	Yes
Would you be willing to review a revision of this manuscript?	Yes

Comments to author: In the manuscript „The influence of platelet-derived concentrates on wound healing" the authors tested the effect of PMC (platelet-mediator concentrate) on proliferation and migration of keratinocytes in vitro as well as the effect of PRP (platelet rich plasma) on chronic wounds in vivo.

In an aging society with an increasing number of patients with chronic wounds, the topic is important and relevant. The study is well conducted and shows interesting results. However, some minor concerns have to be addressed.

Page 5/30, line 6: The authors state that for migration assays 10,000 cells were seeded into 22 square-cm dishes and cultivated with 1.5 ml medium. There is no way 10,000 cells seeded into 22 cm2 will become confluent in 1 day. The same number of cells is seeded into 24-wells for proliferation assays in this manuscript, and a 24-well-plate well has 2 cm2. Also, 1.5 ml is definitely not enough volume to cover the surface. There must be some sort of confusion. Additionally, the authors should revise their manuscript regarding the use of a comma to separate decimals. Also, is 10 x 103 instead of 104 an accepted notation? I have never seen it before.

The description of the proliferation assay method is missing the information that the cells have been detached prior to counting. Without this information, the y-axis labeling for figure 3 (Keratinocytes/mL) makes no sense. Also: is there a reason why the 1%-and the 10%-treatment have their own respective control? It could have been done with one control for both and all results in one diagram.

The manuscript should be revised regarding the language. For example, the sentence "For each patient, every time of measurement and for the control groups 3 replica each were created." (page 4, line 54) is somewhat confusing. There are several occasions with somewhat confusing sentences. Another example: on page 4, line 56 it must read "trypan blue", not "tryptaneblue".

Reviewer 2: anonymous

Date received: 13-Jun-2022

Reviewer recommendation: **Return to author for major modifications**

Reviewer overall scoring: **Low**

Assessment Form scores: 5 = High/Yes; 3 = Medium/Adequate; 1 = Low

Is the subject area appropriate for the journal			3		
Does the title clearly reflect the paper's content?				2	
Does the abstract clearly reflect the paper's content				2	
Do the keywords clearly reflect the paper's content?			3		
Does the introduction present the problem clearly?				2	
Are the results/ conclusions justified?					1
How comprehensive and up-to-date is the subject matter presented?				2	
How adequate is the data presentation?					1
Are units and terminology used correctly?				2	
Is the number of cases adequate?					1
Are the experimental methods/ clinical studies adequate?					1
Is the length appropriate in relation to the content?				2	
Does the reader get new insights from the article?				2	
Please rate the practical significance.			3		
Please rate the accuracy of methods.					1
Please rate the statistical evaluation and quality control.					1

Please rate the appropriateness of the figures and tables.				2	
Please rate the appropriateness of the references.				2	
Please evaluate the writing style and use of language.		4			
Please judge the overall scientific quality of the manuscript.				2	
Are the methods used worthy of reproduction in greater deal?	Yes				
Would you be willing to review a revision of this manuscript?	Yes				

Comments to author:

General comments

Title:

- misleading title , as the content of the manuscript does not at all describe the effects of prp on wound healing, detailed comments in the following text
- Proposal to change the title to: In-vitro effects of platelet-derived concentrates on stored keratinocytes and case series of application to wounds of different etnities to assess wound healing in chronic wounds
- also consider to use the term chronic wounds, as all the patients seem not to heal regulary
- please state the level of evidence for this work
- please state if there is any conflict of interest existing

Abstract

- 49 Results: you write in your abstract a faster wound healing, faster in comparison to what? is there a comparison group here to which the wound healing was faster?
- 49-50 you write about a good functional outcome, how was the functional outcome measured? in the material and methodology part of the abstract you do not write how the functionality was tested.

- 55 Conclusion:

Misleading conclusion

You write that it is a good additive therapy, but in the cases described, isn't it rather the attempt to treat the wounds after unsuccessful surgical treatment ?

Introduction

Page 2, 54- Page 3, 3-6

this belongs in the methodology section, the introduction could be a good place to list the pros and cons of the various processing options.

Page 3, 27-32

"Aim of the study was to analyze the effect of platelet concentrates on the healing rate
and blood perfusion of problematic chronic wounds as well as the patient's quality of life
during healing process. "

this sentence makes no sense at all and leaves the reader in a state of confusion.

-it is now written about perfusion and before that it is not mentioned at all what role perfusion plays. Is the work focussing on wounds that have developed due to poor perfusion, as is the case with arterial occlusive disease?

- in the abstract you also mention the good functional outcome, how does the manuscript intend to answer this?

Materials and Methods

in vitro testing

- how did you isolate the cells from the tissue resectate and how long were the cells stored?

Have the different cell series been stored for different lengths of time?

in vivo testing

p 5 , 49-52 "A prospective case series with 5 complex soft tissue defects was performed where initial

wound closure had failed. A non-healing wound state of over 21 days was required."

-as you write here it was a prospective case series, please add in the title as described before

p5, 49-52 "A prospective case series with 5 complex soft tissue defects was performed where initial wound closure had failed."
- the inclusion criteria are very confusing, were they complex soft tissue defects? or were they chronic wounds? how were the patients selected? was there blinding?

one really important piece of information is missing here and leaves the reader in the dark. how exactly was a chronic wound defined? what were the authors' criteria for defining chronic wounds?

Moreover, it is a composite selection of patients without a comparison group or even of comparative wounds within the individual patient that were not treated, for example.

I recommend researching the literature again and also listing the controversial literature on the definition in the introduction.

p8-9 Follow-up care
how did you standardize the measurement, it is known that in laser doppler imaging blood pressure and temperature of the affected area affect the measurement results?

Authors' Response to Reviewer Comments

Date received: 21-Jun-2022

Response to reviewer 1

- *Page 5/30, line 6: The authors state that for migration assays 10,000 cells were seeded into 22 square-cm dishes and cultivated with 1.5 ml medium. There is no way 10,000 cells seeded into 22 cm will become confluent in 1 day. The same number of cells is seeded into 24-wells for proliferation assays in this manuscript, and a 24-well-plate well has 2 cm. Also, 1.5 ml is definitely not enough volume to cover the surface. There must be some sort of confusion*

→ Thank you very much for the detailed review and the valid hint! After reviewing the lab book, we actually found a mistake in the reporting of the numbers in the manuscript. While 10^4 cells were present in each ml, we used about 15ml to cover the whole surface of the dish. The amount of cells was updated accordingly.

- *Additionally, the authors should revise their manuscript regarding the use of a comma to separate decimals. Also, is 10 x 10³ instead of 10⁴ an accepted notation? I have never seen it before.*

→ We revised all numbers.

- *The description of the proliferation assay method is missing the information that the cells have been detached prior to counting. Without this information, the y-axis labeling for figure 3 (Keratinocytes/mL) makes no sense.*

→ Thank you for the hint, this paragraph was added to better understand the used method: "Medium in the well was removed and the cells washed with PBS w/o. Subsequently, trypsin 0.05%/EDTA 0.02% (Cytogen®, Greven, Germany) was added to the cells and left on for about 2 minutes. [...]"

- *Also: is there a reason why the 1%-and the 10%-treatment have their own respective control? It could have been done with one control for both and all results in one diagram.*

→ The experiments using 1% PMC and 10% PMC were done one after the other and not in parallel dishes. We wanted to have better comparisons in each group, so we decided to do new control groups (though both controls shown are almost identical in number of cells). We think, that the difference between 1% PMC and 10% PMC is better displayed this way. If desired, the data can be edited into one diagram.

- *The manuscript should be revised regarding the language. For example, the sentence "For each patient, every time of measurement and for the control groups 3 replica each were created." (page 4, line 54) is somewhat confusing. There are several occasions with somewhat confusing sentences. Another example: on page 4, line 56 it must read "trypan blue", not "tryptaneblue"*

→ Thank you again for the advice. We revised the manuscript once more for possible spelling mistakes or translation mistakes to make reading easier.

Response to reviewer 2

- *Title: - misleading title , as the content of the manuscript does not at all describe the effects of prp on wound healing, detailed comments in the following text - Proposal to change the title to: In-vitro effects of platelet-derived concentrates on stored keratinocytes and case series of application to wounds of different entities to assess wound healing in chronic wounds*

→ Title changed to "Platelet-derived concentrates influence human keratinocyte proliferation *in vitro* and induce wound healing in a case series of chronic wounds of different entities *in vivo*"

- *also consider to use the term chronic wounds, as all the patients seem not to heal regulary*

→ "Chronic soft tissue defect" was replaced by "chronic wounds" where the meaning was identical

- *please state the level of evidence for this work*

→ This paragraph was added into the methods section: "The Agency for Healthcare Research and Quality (AHRQ) finds all case series to be Level V, whereas in the definition of the Centre for Evidence-Based Medicine (CEBM) all case studies are Level 4."

- *please state if there is any conflict of interest existing*

→ There is no conflict of interest as stated in the Template for Ethical and Legal Declarations (separate file to the main document).

- *Abstract - 49 Results: you write in your abstract a faster wound healing, faster in comparison to what? is there a comparison group here to which the wound healing was faster?*

→ We changed the language to "initiation of wound closure", since the patients had almost no wound healing prior to PRP injection. Only after injection did the healing rate of each patient improve, so wound closure could be achieved. We tried to show this change in figure 6. There was no comparison group due to ethical reasons, as this pilot study was designed as individual healing attempt for each patient.

- *49-50 you write about a good functional outcome, how was the functional outcome measured? in the material and methodology part of the abstract you do not write how the functionality was tested.*

→ We measured the blood flow after PRP injection to show better vascularization, which eased the surgical procedure of split-thickness skin grafting. Since the use of "functionality" in this context is misunderstanding, we rephrased the statements to: "[...] This effect is mediated by an increase in the proliferation of keratinocytes - as could be shown in the *in vitro* tests - and the improved blood flow to the edges of the wound. This leads to a simplification of the surgical wound closure [...]".

- *55 Conclusion: Misleading conclusion. You write that it is a good additive therapy, but in the cases described, isn't it rather the attempt to treat the wounds after unsuccessful surgical treatment?*

→ Thank you for the suggestion. The conclusion was reworded accordingly.

- *Introduction. Page 2, 54- Page 3, 3-6 this belongs in the methodology section, the introduction could be a good place to list the pros and cons of the various processing options.*

→ The above-mentioned paragraph was moved to the methodology section as advised. The Pros and Cons of the processing options are reviewed in detail in the discussion: "There are two main types of platelet concentrates: Platelet Rich Plasma (PRP) and Platelet Mediator Concentrate (PMC). [...]"

- *Page 3, 27-32 "Aim of the study was to analyze the effect of platelet concentrates on the healing rate and blood perfusion of problematic chronic wounds as well as the patient's quality of life during healing process". This sentence makes no sense at all and leaves the reader in a state of confusion.-it is now written about perfusion and before that it is not mentioned at all what role perfusion plays. Is the work focussing on wounds that have developed due to poor perfusion, as is the case with arterial occlusive disease? - in the abstract you also mention the good functional outcome, how does the manuscript intend to answer this?*

→ Thank you again for the detailed reading. The paragraph was revised accordingly to give the reader a better understanding.

- *Materials and Methods in vitro testing*
  - how did you isolate the cells from the tissue resectate and how long were the cells stored? Have the different cell series been stored for different lengths of time?

→ We had a separate supplementary text, where the local protocol was summarized. We initially didn't add this to the manuscript due to the required word count limit. The required paragraph was added to the methodology section, page 4. The samples were stored for several weeks, though the exact time is no longer comprehensible in retrospection.

- *in vivo testing p 5 , 49-52 "A prospective case series with 5 complex soft tissue defects was performed where initial wound closure had failed. A non-healing wound state of over 21 days was required."-as you write here it was a prospective case series, please add in the title as described before.*

→ Thank you for the suggestion, the title was revised.

- *p5, 49-52 "A prospective case series with 5 complex soft tissue defects was performed where initial wound closure had failed." - the inclusion criteria are very confusing, were they complex soft tissue defects? or were they chronic wounds? how were the patients selected? was there blinding?*

→ All patients who came to our clinic and had chronic wounds (as defined by non healing wound >21 days and failed surgical intervention) were screened for participation, as can be seen in the CONSORT flow chart. The patients with chronic wound healing included in this study all had complex defects, though that was not a requirement. It took about a year to recruit 5 patients. These were all hospitalized due to their wound healing disorder. Therefore, there was no blinding.

- *one really important piece of information is missing here and leaves the reader in the dark. how exactly was a chronic wound defined? what were the authors' criteria for defining chronic wounds?*

→ A basic definition was added into the introduction: "Since the definition of when a wound becomes chronic varies between authors, a definition based on the appropriate timing of wound stage is more suitable." The definition used in the manuscript was added into the methods section. "Chronic wound was defined by a non-healing wound state of over 21 days, refractory to surgical or conservative therapy."

- *Moreover, it is a composite selection of patients without a comparison group or even of comparative wounds within the individual patient that were not treated, for example.*

→ There is no control group, partly due to ethical reasons in the use of PRP as individual healing attempt. To have a better comparison, we tried to show in figure 4 and figure 6, the individual change of healing rate for each patient over time.

- *I recommend researching the literature again and also listing the controversial literature on the definition in the introduction.*

→ As stated, there is no uniform definition on chronic wounds. We added a short paragraph on the difficulty of finding a common definition in the introduction (page 2) as well as a more detailed description in the discussion (page 20). An overview on the topic was done by Gould et al., 2015; which is cited for further reference.

- *p8-9 Follow-up care, how did you standardize the measurement, it is known that in laser doppler imaging blood pressure and temperature of the affected area affect the measurement results?*

→ We tried to standardization our method by showing the following procedures: “The distance between scan head and wound was approximately 20cm. The scan was performed at room temperature in a darkened room during routine dressing change, adjacent skin was covered by sterile dark green cloth to minimize light reflection. The patient was asked not to move or talk during scans and was not talked to, to minimize artificial blood flow.” By trying to replicate the same conditions, we tried to minimalize external influence. We compared the average changes in tissue perfusion over time (as shown in figure 7) instead of absolute Perfusion Units to minimalize individual fluctuations.

Thank you aging for reviewing this manuscript. We hope, that we could answer all comments to the editors and reviewers satisfaction. If further adjustments are necessary, we will be glad to receive additional comments.

Reviewers’ Comments to the Revised Manuscript

Reviewer 2: anonymous

Date received: 08-Jul-2022
Reviewer recommendation: **Return to author for major modifications**
Reviewer overall scoring: **Low**

Assessment Form scores: 5 = High/Yes; 3 = Medium/Adequate; 1 = Low

Is the subject area appropriate for the journal				2	
Does the title clearly reflect the paper's content?		4			
Does the abstract clearly reflect the paper's content				2	
Do the keywords clearly reflect the paper's content?		4			
Does the introduction present the problem clearly?					1
Are the results/ conclusions justified?					1
How comprehensive and up-to-date is the subject matter presented?			3		
How adequate is the data presentation?				2	
Are units and terminology used correctly?				2	
Is the number of cases adequate?					1
Are the experimental methods/ clinical studies adequate?				2	
Is the length appropriate in relation to the content?				2	
Does the reader get new insights from the article?				2	
Please rate the practical significance.				2	
Please rate the accuracy of methods.				2	
Please rate the statistical evaluation and quality control.				2	
Please rate the appropriateness of the figures and tables.			3		
Please rate the appropriateness of the references.			3		

Please evaluate the writing style and use of language.				2	
Please judge the overall scientific quality of the manuscript.				2	
Are the methods used worthy of reproduction in greater deal?	Yes				
Would you be willing to review a revision of this manuscript?	Yes				

Comments to author: Thank you for submitting your revision.
Please read again the authors' notes for the submission of manuscripts. The work exceeds the maximum word count.

The treatments you describe would be interesting to see as illustrations to assess the wound before the operations, after the unsuccessful operations and after experimental injection therapy to heal. Unfortunately, the work does not allow insight into these treated wounds. It would also be valuable to assess how long the healed wounds remained closed.

If the conclusion of the work is that healing can take place by improving blood circulation, then regular wounds should also heal when blood circulation is optimised, even if functional structures are exposed and they are large multi-layer wounds. This is not addressed.

Introduction:
13-16 " If an initial injury does not heal after the expected amount of time and appropriate treatment, it is considered a chronic wound. "

This is one of the first sentences in the introduction, which already imprecisely gives the definition
One definition may be: A chronic wound is one that has failed to progress through the phases of healing in an orderly and timely fashion and has shown no significant progress toward healing in 30 days
but there are other definitions that need to be included in the introduction to explain the problem to the reader. Discuss those different definitions so the reader can understand.

In general, the introduction does not seem precise enough and lacks in detail.

In my opinion, there is no such thing as "the chronic wound", but a distinction must be made here according to the etnity. is it a chronic pressure ulcer or is it a chronic vascular ulceration? why should a chronic pressure ulcer heal if only the circulation is improved but the basic problem remains?

30-60 “It has been shown that chronic wounds, for example burn wounds, lead to psychological distress even many years after initial trauma (Berg et al., 2016).”

the psychological stress after an initial burn trauma can probably not be compared to chronic wounds here. the acute trauma event of a burn has a completely different meaning than a chronic wound of a specific ethnicity.

35-39 “... Quality of Life questionnaire, developed in Hamburg, Germany (Blome, Baade, Debus, Price, & Augustin, 2014) was used, to evaluate the quality of life in patients before and after surgical procedures. “

i don't understand, before and after which surgical procedures? the surgical rehabilitation and reconstruction failed, didn't it? do you rather mean the injection with platelet concentrates?

Material and Methods

P3 56-59, p4 3-6 “ Due to this aspect two protocols were examined. We decided to use platelet mediator concentrate (PMC) for in vitro testing, since platelets do not activate in a culture flask the way they do around complex human tissue. For the clinical study we used platelet rich plasma (PRP) since it is easier and faster to prepare during surgery .”

what is the difference in the components?

P6 "A prospective case series with five chronic wounds was performed. A chronic wound was defined by a non-healing wound state of over 21 days, refractory to surgical or conservative therapy."

What surgical procedures were done before?

It is completely unclear how long these wounds have existed, how they were treated and what the condition of the wound was at the time of the injection.

P14 29-49
"(3) Wound preparation and STSG harvesting Each patients´ wounds were treated before by mechanical surgical debridement and extensive cleansing using 0,9% saline to remove all necrotic or potentially infectious tissue. The wound margins were injected with beforehand prepared PRP (10mL) using a sterile syringe with a 22-gauge needle. The wound was then covered with foam dressing, which stayed in place for 3 days to ensure manipulation free healing. PRP application was performed on days 0, 7 and 14. If coverage of the wound was necessary/possible, split skin grafts were harvested from the anterior tight using a Padgett dermatome, approximately 0.2mm in width, and secured in place using staples."

it is completely unclear what was done here. the wound edges were treated with prp. it is described before that functional structures such as tendons or bones are partially exposed. then the prp injection is repeated in the wound edges on the 0,7,14 day and if necessary split skin is then transplanted. this is incomprehensible, was there suddenly well perfused tissue on the functional structures? although only the wound edges were injected? how can this happen in large wounds?

In this study, there is neither a systematic selection cohorts, nor a standardised treatment nor a standardised evaluation. perfusion depends on blood pressure, positioning of the patient and body temperature, possibly the temperature of the affected extremity. simply holding the laser doppler on a wound is not a sound scientific evaluation. the lack of clinical images to assess the wounds is also not mentioned here. the reader is left in the dark as to what the wounds were in the first place and how they presented themselves. The ability to reproduce experiments is a defining principle of science.

- Please add patient images to the publication
- the introduction and discussion must take into account the above points
- please list the failed surgical reconstructions for each patient.

Authors' Response to Reviewer Comments an the Revised Manuscript

Date received: 11-Jul-2022

Response to reviewer 2

We thank the reviewer again for the questions and comments. We answered each point and tried to improve our manuscript according to the given advice. The text was revised in the mentioned sections. All changes were highlighted in red.

- *„The treatments you describe would be interesting to see as illustrations to assess the wound before the operations, after the unsuccessful operations and after experimental injection therapy to heal. Unfortunately, the work does not allow insight into these treated wounds. It would also be valuable to assess how long the healed wounds remained closed.*
  - Please add patient images to the publication"

We gladly added example pictures from the photographic documentation of our patients in figure 2 and figure 3. Our photo documentation started at day 0 through day 21, so unfortunately there is no photographs prior to study enrollment respectively before any surgical procedure. There is however documentation on the wound size in surgery protocols, which are illustrated in figure 6. After initiation of wound closure all wounds remained closed respectively the wound area did not increase again.

- *"If the conclusion of the work is that healing can take place by improving blood circulation, then regular wounds should also heal when blood circulation is optimized, even if functional structures are exposed and they are large multi-layer wounds. This is not addressed."*

In our small study group, we tried to show that by PRP injection microcirculation can be improved in chronic wounds, which leads the wound out of the arrested state of inflammation. Our hypothesis cannot be generalized or applied to regular wounds since the physiology of healing is not impaired and other factors may be more essential (e.g. the weakest link in the chain is different for chronic wounds and acute wounds).

- *Introduction:*
  13-16 " If an initial injury does not heal after the expected amount of time and appropriate treatment, it is considered a chronic wound. This is one of the first sentences in the introduction, which already imprecisely gives the definition. One definition may be: A chronic wound is one that has failed to progress through the phases of healing in an orderly and timely fashion and has shown no significant progress toward healing in 30 days, but there are other definitions that need to be included in the introduction to explain the problem to the reader. Discuss those different definitions so the reader can understand. In general, the introduction does not seem precise enough and lacks in detail.

Thank you very much for the correction, we changed mentioned paragraph and revised the introduction accordingly to be more precise and added some details for further explanation.

- *In my opinion, there is no such thing as "the chronic wound", but a distinction must be made here according to the entity. is it a chronic pressure ulcer or is it a chronic vascular ulceration? why should a chronic pressure ulcer heal if only the circulation is improved but the basic problem remains?*

Our study group is very heterogeneous, which is ultimately due to the recruitment directly from the daily clinical routine. Due to the limited cases, the authors cannot draw general conclusions about a specific wound etiology. We tried to give an overview of the wounds analyzed in table 1 and further addressed this limitation in the discussion section.

For the specific problem of chronic pressure ulcer, in our study patient °5 had such a lesion. The basic problem (venous congestion) has already been treated, however, the wound remained even after resolving oedema. In this context, our study analyzed just the effect on wound healing, assuming that the triggering causes have already been treated in advance. Certainly, further studies - especially for specific entities - would be interesting and would further specify the clinical use of PRP.

- *30-60 "It has been shown that chronic wounds, for example burn wounds, lead to psychological distress even many years after initial trauma (Berg et al., 2016)." The psychological stress after an initial burn trauma can probably not be compared to chronic wounds here. the acute trauma event of a burn has a completely different meaning than a chronic wound of a specific ethnicity.*

Thank you for pointing this out, we changed the sentence and source to a more general definition especially for chronic wounds.

- *35-39 "… Quality of Life questionnaire, developed in Hamburg, Germany (Blome, Baade, Debus, Price, & Augustin, 2014) was used, to evaluate the quality of life in patients before and after surgical procedures. "I don't understand, before and after which surgical procedures? the surgical rehabilitation and reconstruction failed, didn't it? do you rather mean the injection with platelet concentrates?*

Thanks again for the detailed reading. Indeed, we meant the PRP injection. The statement was corrected for better understanding.

- *Material and Methods*
  P3 56-59, p4 3-6 "Due to this aspect two protocols were examined. We decided to use platelet mediator concentrate (PMC) for in vitro testing, since platelets do not activate in a culture flask the way they do around complex human tissue. For the clinical study we used platelet rich plasma (PRP) since it is easier and faster to prepare during surgery ." what is the difference in the components?

We revised this paragraph and moved it in the introduction section with a more detailed explanation to the differences and why each substance was used in different settings.

- *P6 "A prospective case series with five chronic wounds was performed. A chronic wound was defined by a non-healing wound state of over 21 days, refractory to surgical or conservative therapy." What surgical procedures were done before? It is completely unclear how long these wounds have existed, how they were treated and what the condition of the wound was at the time of the injection.*
  - please list the failed surgical reconstructions for each patient

Thank you very much for the advice. We added a column into table 1 to show all failed surgical procedures as well as negative pressure wound therapy prior to study enrollment. The time course of procedures is also illustrated in figure 6. We hope, this can help the reader to better understand the long standing clinical problem of these patients' wounds.

- *P14 29-49*
  "(3) Wound preparation and STSG harvesting Each patients´ wounds were treated before by mechanical surgical debridement and extensive cleansing using 0,9% saline to remove all necrotic or potentially infectious tissue. The wound margins were injected with beforehand prepared PRP (10mL) using a sterile syringe with a 22-gauge needle. The wound was then covered with foam dressing, which stayed in place for 3 days to ensure manipulation free healing. PRP application was performed on days 0, 7 and 14. If coverage of the wound was necessary/possible, split skin grafts were harvested from the anterior tight using a Padgett dermatome, approximately 0.2mm in width, and secured in place using staples."It is completely unclear what was done here. the wound edges were treated with prp. It is described before, that functional structures such as tendons or bones are partially exposed. then the prp injection is repeated in the wound edges on the 0,7,14 day and if necessary split skin is then transplanted. this is incomprehensible, was there suddenly well perfused tissue on the functional structures? although only the wound edges were injected? how can this happen in large wounds?

We are glad, that our results in this regard are positively surprising. The wounds were assessed by experienced surgeons. As stated above, as soon as sufficient granulation tissue was available, the indication for skin grafting was given. We saw the formation of granulation tissue as combined effect from PRP injection, which improved epithelialization and vascularization. In fact, sufficient formation of granulation tissue was shown only after PRP injection (especially after the first week). Before that, a "simple" coverage was not possible according to clinical aspects. PRP injection considerably simplified the necessary intervention, which we have tried to show in Table 3 by using the "Ease of wound closure scale".

- *In this study, there is neither a systematic selection cohort, nor a standardized treatment nor a standardized evaluation. perfusion depends on blood pressure, positioning of the patient and body temperature, possibly the temperature of the affected extremity. simply holding the laser doppler on a wound is not a sound scientific*

> *evaluation. the lack of clinical images to assess the wounds is also not mentioned here. the reader is left in the dark as to what the wounds were in the first place and how they presented themselves. The ability to reproduce experiments is a defining principle of science.*
> *The introduction and discussion must take into account the above points*

Thank you again for the detailed reviewing our manuscript. We revised the discussion to further point out the limitations of methodology and results of our pilot study. We tried to point out, how difficult the clinical situation in each patient was and that PRP injection was an individual attempt for wound healing. We hope that our approach encourages other clinicians to use PRP in chronic wounds.

Comments by the Editor-in-Chief to Revised Submission

All reviewer comments were addressed adequately and the manuscript should be published in its present stage.
